# Supplementary material for: Comparison of Virulence-Factor-Encoding Genes and Genotype Distribution amongst Clinical Pseudomonas aeruginosa Strains
Source: Int J Mol Sci. 2023 Jan 9;24(2):1269. doi: 10.3390/ijms24021269 (PMC9863696; doi:10.3390/ijms24021269)
Supplement: Supplementary file 1 [file ijms-24-01269-s001.zip › ijms-2085393-supplementary.pdf]

Comparison of virulence factor-encoding genes and genotypes distribution amongst clinical *Pseudomonas aeruginosa* strains

Tomasz Bogiel[1*], Dagmara Depka[1], Stanisław Kruszewski[2], Adrianna Rutkowska[3], Piotr Kanarek[4], Mateusz Rzepka[1], Jorge H Leitão[5*], Aleksander Deptuła[6] and Eugenia Gospodarek-Komkowska [1]

[1] Microbiology Department, Ludwik Rydygier Collegium Medicum in Bydgoszcz, Nicolaus Copernicus University in Toruń, 85-094 Bydgoszcz, Poland and Clinical Microbiology Laboratory, doctor Antoni Jurasz University Hospital No. 1 in Bydgoszcz, Poland

[2] Institute of Forensic Genetics Ltd., Bydgoszcz, Poland

[3] Medicover Integrated Clinical Services, Bydgoszcz, Poland

[4] Department of Microbiology and Food Technology, Faculty of Agriculture and Biotechnology, Bydgoszcz University of Science and Technology, Bydgoszcz, Poland

[5] Department of Bioengineering, IBB-Institute for Bioengineering and Biosciences, Instituto Superior Técnico, Universidade de Lisboa, Av. Rovisco Pais, 1049-001 Lisboa, Portugal. Associate Laboratory, i4HB-Institute for Health and Bioeconomy, Instituto Superior Técnico, Universidade de Lisboa, Av. Rovisco Pais, 1049-001 Lisboa, Portugal

[6] Department of Propaedeutics of Medicine and Infection Prevention, Ludwik Rydygier Collegium Medicum in Bydgoszcz, Nicolaus Copernicus University in Torun, 85-094 Bydgoszcz, Poland

* Correspondence (T.B.): e-mail: t.bogiel@cm.umk.pl, telephone number: +48 52 585 44 80, (J.H.L): e-mail: jorgeleitao@tecnico.ulisboa.pt, telephone number: +351 218417688

Table S1. Distribution of the genes and genotypes with respect to antimicrobial susceptibility profiles of the multidrug–sensitive *P. aeruginosa* strains included in the study (*n* = 74).

Strain number (*n* = 74)	*lasB*	*plC N*	*plC H*	*exoU*	*nan1*	*pilA*	*pilB*	Genotype	*n*	%
11	+	+	+	+	+	-	+	I-S	1	1.4
8	+	+	+	+	+	-	-	II-S	10	13.5
14	+	+	+	+	+	-	-			
32	+	+	+	+	+	-	-			
38	+	+	+	+	+	-	-			
59	+	+	+	+	+	-	-			
62	+	+	+	+	+	-	-			
64	+	+	+	+	+	-	-			
66	+	+	+	+	+	-	-			
72	+	+	+	+	+	-	-			
73	+	+	+	+	+	-	-			
12	+	+	+	+	-	-	-	III-S	15	20.3
23	+	+	+	+	-	-	-			
26	+	+	+	+	-	-	-			
27	+	+	+	+	-	-	-			
29	+	+	+	+	-	-	-			
36	+	+	+	+	-	-	-			
47	+	+	+	+	-	-	-			
49	+	+	+	+	-	-	-			
50	+	+	+	+	-	-	-			
55	+	+	+	+	-	-	-			
57	+	+	+	+	-	-	-			
58	+	+	+	+	-	-	-			
70	+	+	+	+	-	-	-			
80	+	+	+	+	-	-	-			
83	+	+	+	+	-	-	-			
63	+	+	+	-	+	+	-	IV-S	1	1.4
42	+	+	+	-	+	-	-	V-S	6	8.1
75	+	+	+	-	+	-	-			
44	+	+	+	-	+	-	-			
52	+	+	+	-	+	-	-			
60	+	+	+	-	+	-	-			
65	+	+	+	-	+	-	-			
25	+	+	+	-	-	+	-	VI-S	2	2.7
31	+	+	+	-	-	+	-			
3	+	+	+	-	-	-	-	VII-S	22	29.7
13	+	+	+	-	-	-	-			
16	+	+	+	-	-	-	-			
18	+	+	+	-	-	-	-			
19	+	+	+	-	-	-	-			
24	+	+	+	-	-	-	-			
28	+	+	+	-	-	-	-			
30	+	+	+	-	-	-	-			
33	+	+	+	-	-	-	-			
34	+	+	+	-	-	-	-			
37	+	+	+	-	-	-	-			
40	+	+	+	-	-	-	-			

41	+	+	+	-	-	-	-			
48	+	+	+	-	-	-	-			
51	+	+	+	-	-	-	-			
53	+	+	+	-	-	-	-			
61	+	+	+	-	-	-	-			
67	+	+	+	-	-	-	-			
71	+	+	+	-	-	-	-			
76	+	+	+	-	-	-	-			
79	+	+	+	-	-	-	-			
81	+	+	+	-	-	-	-			
20	+	+	-	+	+	-	-	VIII-S	1	1.4
43	+	+	-	+	-	-	-	IX-S	2	2.7
85	+	+	-	+	-	-	-			
46	+	-	+	+	+	-	-	X-S	2	2.7
78	+	-	+	+	+	-	-			
84	+	-	+	+	-	-	-	XI-S	1	1.4
77	+	-	+	-	+	+	-	XII-S	1	1.4
5	+	-	+	-	+	-	-	XIII-S	1	1.4
15	+	-	-	+	-	-	-	XIV-S	2	2.7
74	+	-	-	+	-	-	-			
45	+	-	-	-	-	-	-	XV-S	1	1.4
21	-	+	-	+	-	-	-	XVI-S	2	2.7
22	-	+	-	+	-	-	-			
7	-	+	-	-	-	-	-	XVII-S	3	4.1
17	-	+	-	-	-	-	-			
39	-	+	-	-	-	-	-			
68	-	-	-	-	-	-	-	XVIII-S	1	1.4
n	68	65	62	36	23	4	1			
%	91.9	87.8	83.8	48.6	31.1	5.4	1.4			
	lasB	***plC N***	***plC H***	***exoU***	***nan1***	***pilA***	***pilB***			

(+) – presence of a particular gene; shaded boxes, (-) – absence of a particular gene.

Table S2. Origin and detailed distribution of the genes and genotypes with respect to antimicrobial susceptibility profiles of the multidrug–resistant *P. aeruginosa* strains included in the study (*n* = 57).

Strain numer (*n* = 57)	*lasB*	*plC N*	*plC H*	*exoU*	*nan1*	*pilA*	*pilB*	Genotype	*n*	%
12	+	+	+	+	+	+	-	I-R	1	1.8
10	+	+	+	+	+	-	-	II-R	8	14.0
58	+	+	+	+	+	-	-			
59	+	+	+	+	+	-	-			
61	+	+	+	+	+	-	-			
71	+	+	+	+	+	-	-			
74	+	+	+	+	+	-	-			
78	+	+	+	+	+	-	-			
82	+	+	+	+	+	-	-			
1	+	+	+	+	-	-	-	III-R	20	35.1
7	+	+	+	+	-	-	-			
13	+	+	+	+	-	-	-			
14	+	+	+	+	-	-	-			
15	+	+	+	+	-	-	-			
18	+	+	+	+	-	-	-			
20	+	+	+	+	-	-	-			
27	+	+	+	+	-	-	-			
29	+	+	+	+	-	-	-			
34	+	+	+	+	-	-	-			
51	+	+	+	+	-	-	-			
63	+	+	+	+	-	-	-			
64	+	+	+	+	-	-	-			
65	+	+	+	+	-	-	-			
66	+	+	+	+	-	-	-			
68	+	+	+	+	-	-	-			
79	+	+	+	+	-	-	-			
81	+	+	+	+	-	-	-			
84	+	+	+	+	-	-	-			
85	+	+	+	+	-	-	-			
6	+	+	+	-	+	+	+	IV-R	1	1.8
35	+	+	+	-	+	-	-	V-R	5	8.8
37	+	+	+	-	+	-	-			
41	+	+	+	-	+	-	-			
50	+	+	+	-	+	-	-			
70	+	+	+	-	+	-	-			
21	+	+	+	-	-	+	-	VI-R	1	1.8
3	+	+	+	-	-	-	-	VII-R	19	33.3
4	+	+	+	-	-	-	-			
5	+	+	+	-	-	-	-			
9	+	+	+	-	-	-	-			
16	+	+	+	-	-	-	-			
17	+	+	+	-	-	-	-			
24	+	+	+	-	-	-	-			
26	+	+	+	-	-	-	-			
28	+	+	+	-	-	-	-			
30	+	+	+	-	-	-	-			

31	+	+	+	-	-	-	-			
32	+	+	+	-	-	-	-			
46	+	+	+	-	-	-	-			
47	+	+	+	-	-	-	-			
53	+	+	+	-	-	-	-			
54	+	+	+	-	-	-	-			
56	+	+	+	-	-	-	-			
60	+	+	+	-	-	-	-			
77	+	+	+	-	-	-	-			
2	+	+	-	+	+	-	-	VIII-R	1	1.8
52	+	+	-	-	-	-	-	IX-R	1	1.8
n	57	57	55	30	16	3	1			
%	100.0	100.0	96.5	52.6	28.1	5.3	1.8			
	lasB	***plC N***	***plC H***	***exoU***	***nan1***	***pilA***	***pilB***			

(+) – presence of a particular gene; shaded boxes, (-) – absence of a particular gene

Table S3. The Spearman's rank correlation coefficient for the particular genes pair with respect to antimicrobial susceptibility profiles of *P. aeruginosa* strains included into the study

(types of correlation: *r* > 0 positive correlation, *r* = 0 lack of correlation, *r* < 0 negative correlation; the strength of relation: < 0.2 – lack of relation, 0.2-0.4 – weak, 0.4- 0.7 – moderate, 0.7-0.9 – quite strong > 0.9 – very strong; the statistically significant differences are marked in red)

MDR (*n* = 57)	*plC H*	*exoU*	*nan1*	*pilA*	*pilB*
plC H	1.000000	0.010050	-0.093066	0.044947	0.025482
exoU	0.010050	1.000000	0.123466	-0.091099	-0.140859
nan1	-0.093066	0.123466	1.000000	0.202458	0.213913
pilA	0.044947	-0.091099	0.202458	1.000000	**0.566947**
pilB	0.025482	-0.140859	0.213913	**0.566947**	1.000000

MDS (*n* = 74)	*lasB*	*plC N*	*plC H*	*exoU*	*nan1*	*pilA*	*pilB*
lasB	1.000000	0.040938	**0.675191**	0.091020	0.199480	0.071007	0.034766
plC N	0.040938	1.000000	**0.284966**	-0.051421	-0.107439	-0.093892	0.043552
plC H	**0.675191**	**0.284966**	1.000000	-0.085245	0.216230	0.105166	0.051491
exoU	0.091020	-0.051421	-0.085245	1.000000	0.164199	**-0.232670**	0.120248
nan1	0.199480	-0.107439	0.216230	0.164199	1.000000	0.097715	0.174285
pilA	0.071007	-0.093892	0.105166	**-0.232670**	0.097715	1.000000	-0.027978
pilB	0.034766	0.043552	0.051491	0.120248	0.174285	-0.027978	1.000000

MDS+MDR (*n* = 131)	*lasB*	*plC N*	*plC H*	*exoU*	*nan1*	*pilA*	*pilB*
lasB	1.000000	0.084851	**0.633358**	0.074704	0.142646	0.052055	0.027280
plC N	0.084851	1.000000	**0.296773**	-0.028106	-0.087160	-0.069654	0.033819
plC H	**0.633358**	**0.296773**	1.000000	-0.046777	0.117148	0.082188	0.043072
exoU	0.074704	-0.028106	-0.046777	1.000000	0.145285	-0.171530	-0.000951
nan1	0.142646	-0.087160	0.117148	0.145285	1.000000	0.142229	**0.191241**
pilA	0.052055	-0.069654	0.082188	-0.171530	0.142229	1.000000	**0.247239**
pilB	0.027280	0.033819	0.043072	-0.000951	**0.191241**	**0.247239**	1.000000

Figures S1-S7. Pictures of the electrophoretic gels showing the amplicons of PCR for the corresponding genes investigated in the present study

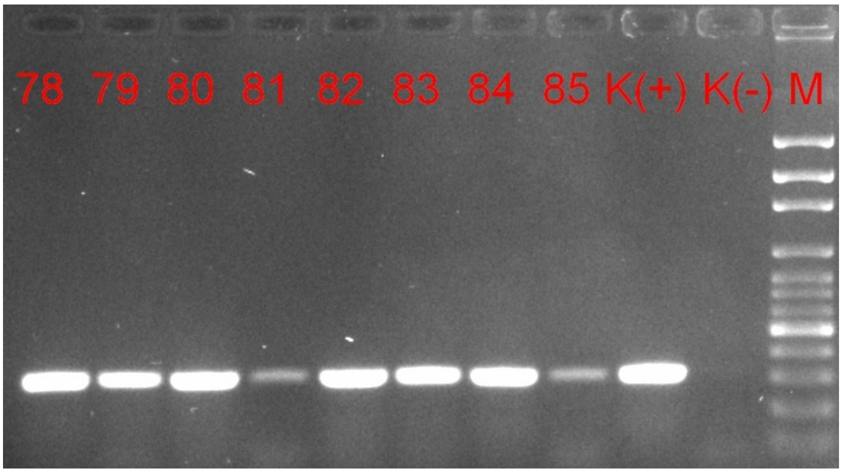

Figure S1 – *lasB* gene (300 bp); M – DNA size marker of 100-3000 bp; numbers – names assigned to a particular strain; K(+) – positive PCR control; K(-) – negative PCR control

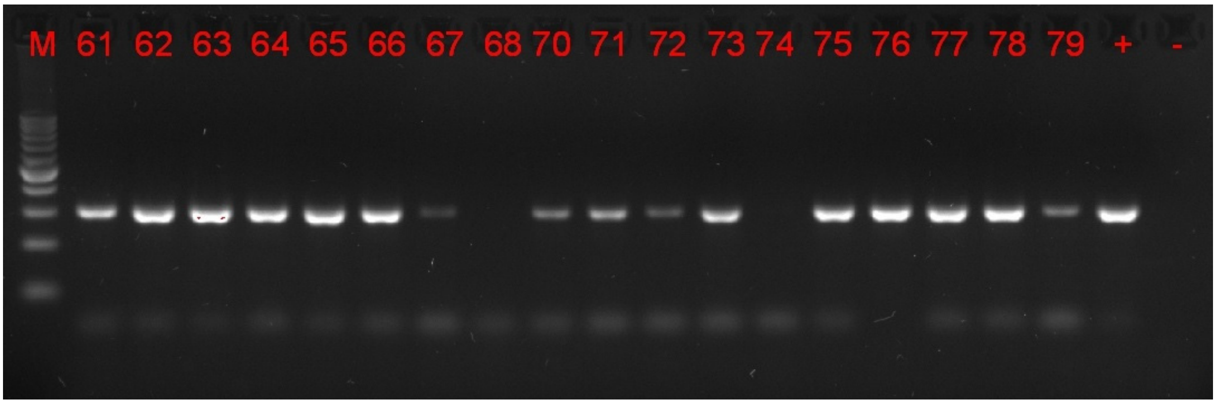

Figure S2 – *plC H* gene (307 bp); M – DNA size marker of 100-1000 bp; numbers – names assigned to a particular strain; (+) – positive PCR control; (-) – negative PCR control

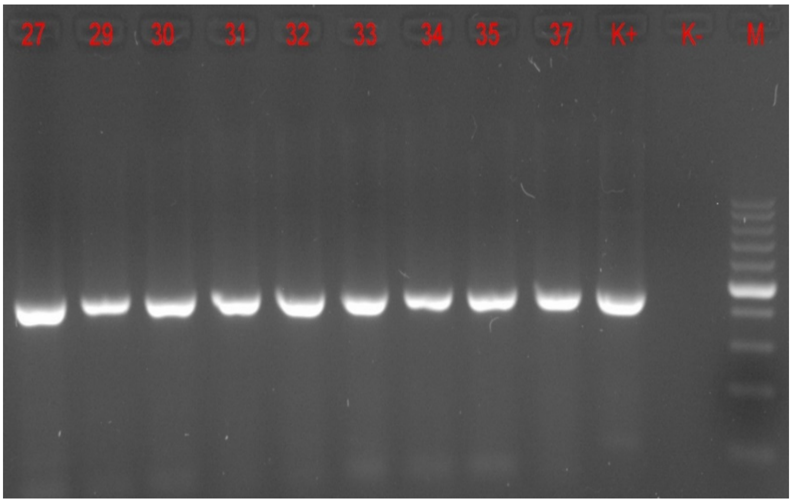

Figure S3 – *plC N* gene (466 bp); M – DNA size marker of 100-1000 bp; numbers – names assigned to a particular strain; K(+) – positive PCR control; K(-) – negative PCR control

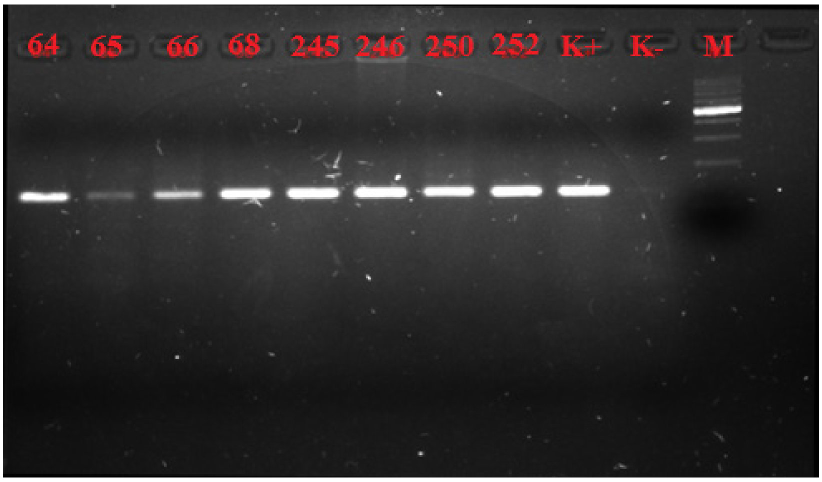

Figure S4 – *exoU* gene (134 bp); M – DNA size marker of 100-1000 bp; numbers – names assigned to a particular strain; K(+) – positive PCR control; K(-) – negative PCR control

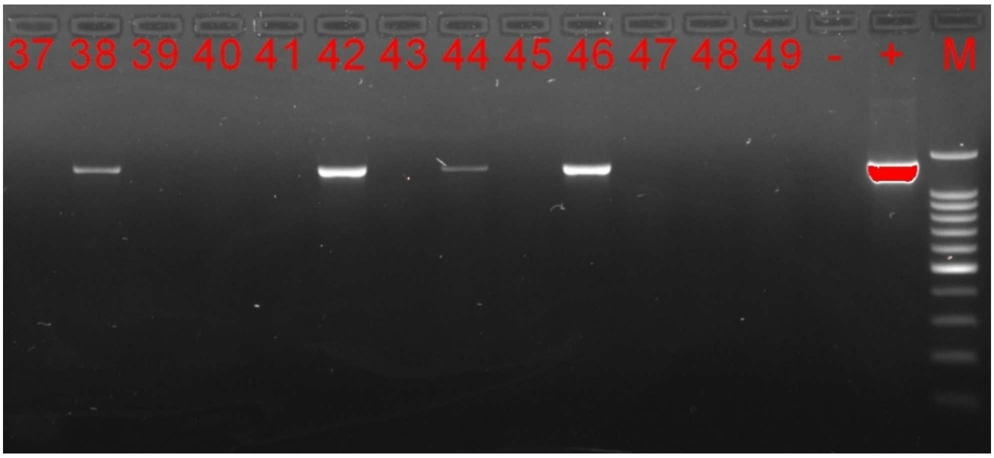

Figure S5 – *nan1* gene (1316 bp); M – DNA size marker of 100-1500 bp; numbers – names assigned to a particular strain; (+) – positive PCR control; (-) – negative PCR control

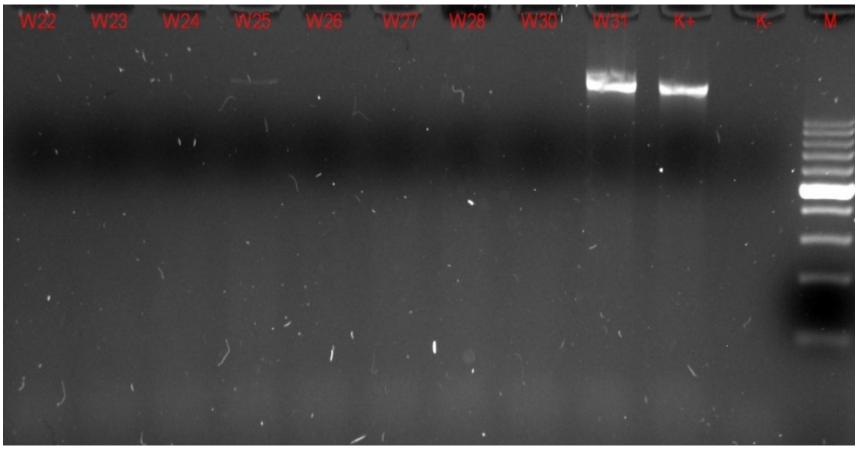

Figure S6 – *pilA* gene (1675 bp); M – DNA size marker of 100-1000 bp; "W"+numbers – names assigned to a particular strain; K(+) – positive PCR control; K(-) – negative PCR control

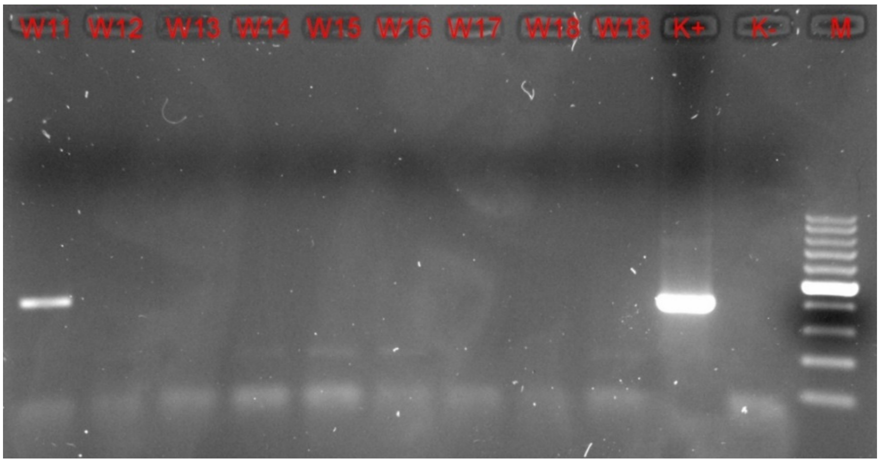

Figure S7 – *pilB* gene (408 bp); M – DNA size marker of 100-1000 bp; "W"+numbers – names assigned to a particular strain; K(+) – positive PCR control; K(-) – negative PCR control
